# Direct trabecular meshwork imaging in porcine eyes through multiphoton gonioscopy

**DOI:** 10.1117/1.JBO.18.3.036009

**Published:** 2013-03-20

**Authors:** Omid Masihzadeh, David A. Ammar, Malik Y. Kahook, Emily A. Gibson, Tim C. Lei

**Affiliations:** a University of Colorado Denver, Department of Ophthalmology, 12800 East 19th Avenue Mailstop 8311, Aurora, Colorado 80045; b University of Colorado Denver, Department of Ophthalmology, 1675 Aurora Court, F731 Aurora, Colorado 80045; c University of Colorado Denver, Department of Bioengineering, 12700 East 19th Avenue Mailstop 8607, Aurora, Colorado 80045; d University of Colorado Denver, Department of Electrical Engineering, Campus Box 110, PO Box 173364, Denver, Colorado 80217-3364

**Keywords:** glaucoma, gonioscopy, trabecular meshwork, aqueous outflow, cornea, sclera, two-photon microscopy

## Abstract

The development of technologies to characterize the ocular aqueous outflow system (AOS) is important for the understanding of the pathophysiology of glaucoma. Multiphoton microscopy (MPM) offers the advantage of high-resolution, label-free imaging with intrinsic image contrast because the emitted signals result from the specific biomolecular content of the tissue. Previous attempts to use MPM to image the murine irido-corneal region directly through the sclera have suffered from degradation in image resolution due to scattering of the focused laser light. As a result, transscleral MPM has limited ability to observe fine structures in the AOS. In this work, the porcine irido-corneal angle was successfully imaged through the transparent cornea using a gonioscopic lens to circumvent the highly scattering scleral tissue. The resulting high-resolution images allowed the detailed structures in the trabecular meshwork (TM) to be observed. Multimodal imaging by two-photon autofluorescence and second harmonic generation allowed visualization of different features in the TM without labels and without disruption of the TM or surrounding tissues. MPM gonioscopy is a promising noninvasive imaging tool for high-resolution studies of the AOS, and research continues to explore the potential for future clinical applications in humans.

## 1 Introduction

In glaucoma, abnormalities in the ocular aqueous outflow system (AOS) can lead to elevated intraocular pressure (IOP) with subsequent death of retinal ganglion cells, resulting in loss of vision.^1^ High-resolution visualization of the AOS, which includes the trabecular meshwork (TM), Schlemm’s canal (SC), and collector channels (CC), would be of great diagnostic value toward understanding the pathophysiology of glaucoma and allow for monitoring of medical and/or surgical interventions that decrease IOP. Current diagnostic techniques used to study the AOS include gonioscopy,^2^ optical coherence tomography (OCT),^3^^–^^8^ and ultrasound biomicroscopy (UBM).^9^^,^^10^ None of these techniques can image with molecular specificity, nor do they have the spatial resolution (∼1 to 5 *µ*m) required to resolve TM structures. Advancement in three-dimensional (3-D) micro–computed tomography (micro-CT) using a synchrotron radiation source can achieve 2-*µ*m voxel resolution, which is adequate for resolving details of the AOS structures including TM, SC, and CC.^11^ However, beam line availability and the associated costs of synchrotron radiation sources prohibit the use of this technique in most laboratories, and the required use of contrast agents prevents its routine clinical use for studying the ocular AOS.

We have previously demonstrated transscleral multiphoton microscopy (MPM) of the TM.^12^^,^^13^ However, because of the thick sclera in the human eye, multiphoton transscleral imaging of the TM is extremely difficult and yields images of only the largest structures of the AOS.^12^ Therefore, new imaging strategies that avoid the transscleral pathway are required to improve on current modalities. One avenue toward this goal is to couple advanced imaging devices to a customized gonioscopic lens.^14^^,^^15^ A gonioscopic lens consists of a transparent material that is placed on the cornea and allows for a direct line of sight to the TM region, something that is not normally possible owing to total internal reflection of light at the cornea–air interface. In this study, we explore the use of a unique MPM setup that leverages a gonioscopic lens for direct transcorneal imaging of the AOS in an intact porcine eye.

## 2 Methods

### 2.1 Porcine Eye and Gonioscopic Lens

**Fig. 1:**
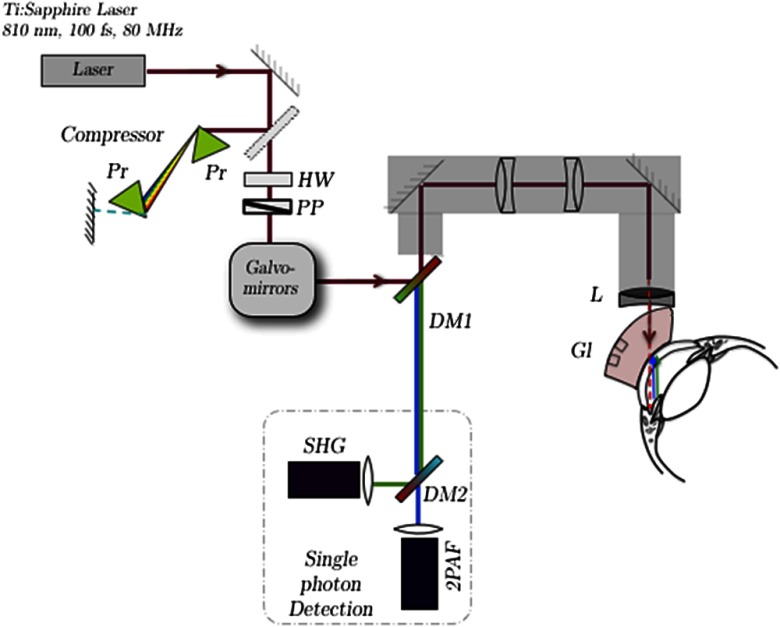
Schematic of the custom multiphoton gonioscopy microscope. The pulsed femtosecond laser beam is first sent through a prism compressor and passed through two galvanometric scanning mirrors for raster scanning at the sample. A custom-made lens relay system is used to convert our inverted microscope (Olympus IX71) to an upright microscope for in situ imaging. The multiphoton microscopy (MPM) signal from the sample is collected back through the microscope objective and separated from the excitation laser light with a dichroic mirror (DM1). An additional dichroic mirror (DM2) is used to spectrally separate the second-harmonic-generation (SHG) and two-photon autofluorescence (2PAF) signals, which are subsequently focused on two single-photon counting photodetectors for data acquisition. Pr, prism; HW, half-wave plate; PP, polarizer; Gl, gonioscopic lens; L, focusing lens.

Enucleated porcine eyes (n=4) immersed in phosphate-buffered saline (PBS) were obtained from Visiontech (Mesquite, Texas) and used within 24 h of death. The extraocular tissues were surgically removed, and each eye was placed in a custom eye holder (Fig. 1) which was mounted on a manipulator allowing translational and rotational positioning of the eye with a high degree of accuracy. A gonioscopic lens (Ocular Hoskins-Barkan Goniotomy-Infant-10 mm Lens; Ocular Instruments, Bellevue, Washington) was secured on a custom holder mounted on a second multidimensional manipulator to allow translational and rotational positioning of the gonioscopic lens. This arrangement allows independent positioning of the gonioscopic lens against the eye to permit optical access of the AOS. Genteal ointment (Alcon, Fort Worth, Texas) was applied between the gonioscopic lens and the eye to provide lubrication as well as an air-free interface between the surface of the cornea and the lens.

### 2.2 Two-Photon Autofluorescence and Second-Harmonic-Generation Microscopy

Multimodal images were obtained using a custom-built MPM system described in detail elsewhere.^16^ Briefly, the MPM system consists of a custom-built laser-scanning microscope with nondescanned detectors. A lens relay converts the microscope from an upright to inverted configuration for easier alignment of targeted tissues. The excitation light was focused through the gonioscopic lens onto the AOS of the eye (Fig. 1), and the emitted two-photon autofluorescence (2PAF) and second-harmonic-generation (SHG) signals were collected through the same optics by the detectors using a dichroic mirror and filter to separate out the excitation light. The excitation laser source was an 80-MHz repetition rate pulsed infrared titanium:sapphire ultrafast laser (Mai Tai HP; Spectra Physics, Santa Clara, California) with a center wavelength of 810 nm. For imaging, the excitation laser was focused with a 25-mm focal length plano-convex singlet lens (LA1951; Thorlabs, Newton, New Jersey) through the gonioscopic lens onto the TM region of the porcine eye. The power at the singlet lens was measured to be 37 mW. However, because of the steep angle of incidence on the surface of the gonioscopic lens, significant Fresnel reflection and scattering occurred, and only ∼85% of the power was transmitted to the surface of the porcine eye as measured with a power meter. There is also further loss of optical power due to scattering by the cornea and the aqueous humor. Therefore, the final total power delivered to the TM region was estimated to be less than ∼25  mW.

The multiphoton signals generated at the laser focus include SHG and 2PAF. The emitted signals were collected in the epi-direction and separated from the excitation laser by a dichroic mirror (long pass at 685 nm, FF685-Di02; Semrock). Any residual excitation laser light was blocked by an additional sputter-coated, high-throughput shortpass two-photon emission filter (ET700sp-2p; Chroma Technology). The SHG and 2PAF signals were spectrally separated with a dichroic mirror (T425lpxr; Chroma Technology) and bandpass filters (HQ400/20m-2p for SHG and HQ575/250m-2p 2PH; Chroma Technology) and detected on two separate channels using high-efficiency photon-counting photomultiplier detectors (H7422P-s40; Hamamatsu).

### 2.3 Image Analysis

Acquired tiled multiphoton images were postprocessed using Matlab (MathWorks, Natick, MA) and ImageJ (http://rsbweb.nih.gov/ij/) software. The size of each individual image was 512×512 pixels. The images were acquired with a 14-*µ*s pixel dwell time and a frame average of two. To image over larger regions of the eye, tiling of several individual scans was performed. In addition, appropriate noise reduction filtering (six-point smoothing function) and thresholding were applied to eliminate noise originating from detector dark counts and optimize visualization. The details of the image-processing techniques and tiling of the final image are described elsewhere.^16^ The effects of tiling could be seen in the reconstructed images; the edges of the individual tiles have discontinuous intensity due to variations in signal intensity across the field of view. This artifact could be eliminated in the future with more sophisticated image reconstruction techniques to correct for the intensity variations observed here.^17^

### 2.4 Histology

In a single experiment, a metal surgical suture was placed through the sclera at the limbal region of the porcine eye. Using the suture as a guide, gonioscopic imaging was performed at the nearby TM region. After the imaging experiment, the porcine eye was preserved in 4% paraformaldehyde/PBS overnight at 4°C and then embedded in paraffin. Sagittal histological sections (6 *µ*m thick) were cut in a region within 200 to 300 *µ*m of the surgical suture. Tissue sections were stained with Mayer’s hematoxylin and eosin Y (H&E Richard-Allan Scientific, Kalamazoo, Michigan). Bright-field imaging was performed using a Nikon Eclipse 80i microscope (Nikon, Melville, New York) equipped with a Nikon D5-Fi1 color camera and a Nikon 4X/Plan Fluor objective lens.

## 3 Results

**Fig. 2:**
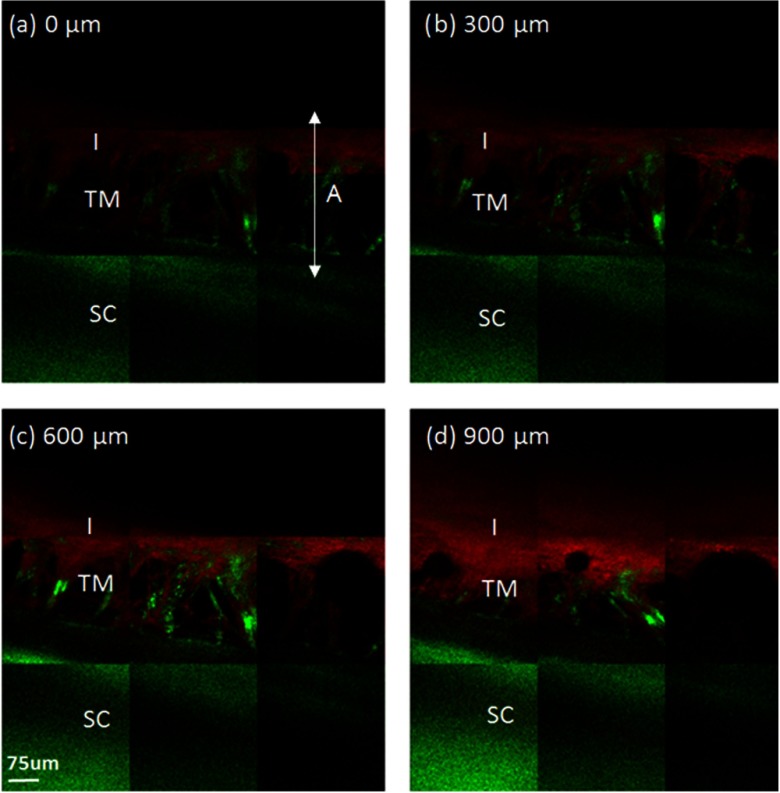
Acquired MPM images of the TM of a porcine eye taken through a gonioscopic lens. Panels 2(a)–2(d) are 3×3 tiles of combined MPM images of the porcine trabecular meshwork (TM) at different depths, where 2(a) is closest to the anterior chamber and 2(d) is farthest away from the anterior chamber. The images are 300 *µ*m apart. SHG signal is displayed in green and 2PAF is displayed in red. A collagen-rich mesh structure (SHG) is observed in all images. The mesh structure is located in a gap that separates two distinct regions: a strong SHG region (sclera) and a strong 2PAF region (iris). SC, sclera; I, iris; A, irido-corneal angle.

2PAF and SHG images were obtained from the AOS of the porcine eye using our custom MPM gonioscopy device (Fig. 2). Multiple porcine eyes (n=4) were imaged over several experimental sessions and across different days, illustrating the reproducibility of our method. Figure 2(a)–2(d) illustrates images at different depths (300 *µ*m apart) within the TM region. The observed 2PAF signal (red) is mainly from NAD(P)H, ﬂavin, collagen, elastin, and melanin; the SHG (green) is from collagen.^18^^,^^19^ The strong 2PAF signal from the iris (I) in Fig. 2 has been shown from our previous work to be primarily from melanin, which exhibits a broad fluorescence band when excited by the infrared femtosecond laser.^20^

**Fig. 3:**
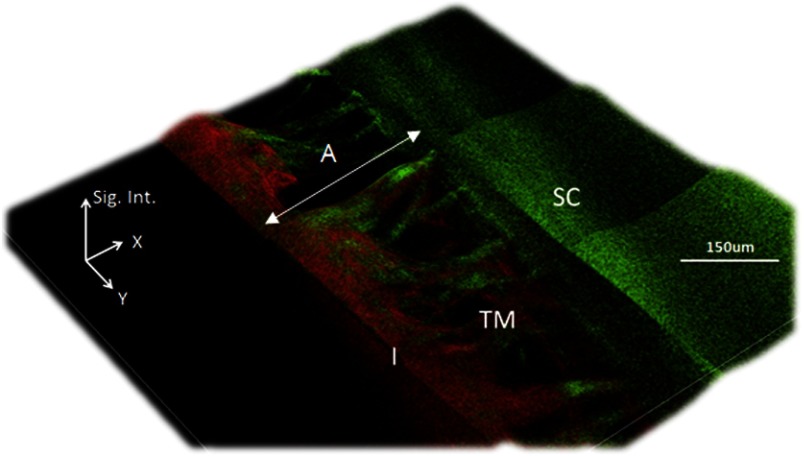
Processed 2.5D pseudo-intensity plot of MPM gonioscopy images. The two-dimensional image of Fig. 2(a) was processed by 2.5D to highlight regions between the collagen fibers lacking signal. The z-axis of the processed 2.5D image is the signal intensity (Sig. Int.) of both the SHG and 2PAF signals (arbitrary units). The dark regions lacking both SHG and 2PAF are assumed to be fluid-filled pores.

Mesh-like structures were observed exhibiting predominantly SHG, indicative of a collagen-based tissue within the TM region.^21^ Areas lacking SHG and 2PAF signal were assumed to be fluid-filled pores, which ranged in size from ∼10 to 50 *µ*m at the deeper layers of the juxtacanalicular TM to as large 150 *µ*m near the TM surface [Fig. 2(a)]. In general, the diameter of these pores became more narrow at deeper depths within the TM [Fig. 2(b) through 2(d)], and these results matched those observed by electron microscopy of the porcine eye.^22^ The TM structures were located between two regions, the first with strong SHG (from the sclera) and the second with strong 2PAF (from the iris). The anterior to posterior width of the mesh structure was ∼200  μm, and the mesh structures were observed circumventing the entire rim of the eye (Fig. 3).

The irregular layers of tissue within the filtering region of TM have been shown by immuno-electron microscopy to be composed of collagen/elastin fibers embedded within extracellular matrix (ECM) and surrounded by a basement membrane of laminin and fibronectin.^23^ The predominant SHG signals in our TM images [Fig. 2(a)–2(d)] confirmed a strong collagen-based meshwork. Furthermore, significant 2PAF signal was noted at the central region of the iridiocorneal angle at a depth indicating the image was within the filtering region of the TM [Fig. 2(b)]. Closer inspection at the TM meshwork showed additional 2PAF near the edges of SHG signals. Owing to the fibrillar nature of the 2PAF, we attribute the 2PAF signal to fluorescence of collagen-associated proteins of the basement membrane or ECM.

**Fig. 4:**
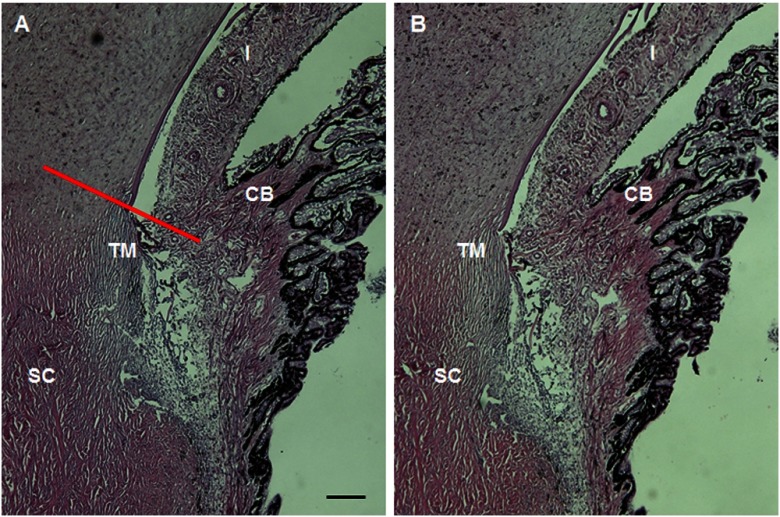
MPM gonioscopy imaging of the TM region of the porcine eye does not appear to damage ocular structures. A histological section of the region of the porcine eye imaged by MPM (b) shows no distortion or photocoagulation of the tissues near the drainage angle compared to a region from the opposite side of the eye (a). The red line in 4(a) shows the approximate imaging plane of Fig. 2(a); 2(b) and 2(c) would be located parallel to this plane traveling deeper into the scleral/TM region. CB, ciliary body. The scale bar represents 200 *µ*m.

Light microscopy images (Fig. 4) were obtained from histological sections of the TM region of the porcine eye previously imaged with our custom MPM gonioscopy system. A metal surgical suture was placed through the sclera adjacent to the imaged section of AOS before fixation of tissue to ensure that histology was performed at the same region exposed to infrared laser light when imaged with the multiphoton system. The histological sections were located within 300 *µ*m of the suture, placing them within the $∼900\times 900\text{-}{\mu \text{m}}^{2}$ area imaged by gonioscopy. Images showed no apparent photocoagulative damage and were similar to histological sections obtained from unperturbed regions of the eye. From this, we conclude that MPM gonioscopic imaging did not cause any thermal damage by laser exposure to the tissues in the AOS.

## 4 Discussion

Conventional single-photon fluorescence microscopy typically requires exogenous fluorophores to label different constituents of the biological sample and therefore is not suitable for longitudinal *in vivo* imaging studies. In addition, optical excitation wavelengths used in single-photon fluorescence microscopy are typically in the visible or ultraviolet wavelengths.^24^ These higher-energy photons undergo greater optical scattering as well as stronger material absorption in biological tissues compared to longer-wavelength infrared light, leading to insufficient optical penetration at depths necessary for imaging the TM.^25^ In contrast, MPM uses near-infrared lasers with extremely short pulse duration ($10^{-13}$ to $10^{-14}\;s$).^26^ Additionally, since multiphoton interactions occur only within the focus of the objective, a thick tissue sample can be imaged through scanning multiple “virtual” sections at various depths. These images can subsequently be reconstructed into 3-D representation. With unique capabilities of subcellular resolution as well as molecular-specific functional imaging through intrinsic optical properties of biological molecules,^27^^–^^29^ MPM would be an excellent addition to the current pool of ophthalmic imaging techniques.^12^^,^^13^^,^^16^^,^^26^^,^^30^^,^^31^

To date, imaging the TM region in an intact human eye using optical microscopy has been challenging. To access the region directly, it is necessary to image through ∼500–600 microns of scleral tissue that is highly scattering.^12^ For example, sclera transmission is measured to be 35% at 804 nm (typical multiphoton excitation wavelength) and is reduced to 5% at ∼400  nm (wavelength of the nonlinear signal emitted by the sample). Even at longer excitation wavelengths (1064 nm), the transmission is only about 53%.^32^ An additional difficulty of transscleral imaging is the distortion of the laser focus when traversing through the scattering media, reducing the resolution obtainable from the technique. The images from our previous work using MPM with infrared excitation light to penetrate through the sclera demonstrate these issues.^12^ Whereas this method was able to resolve the scleral spur along with CC and SC, the detailed mesh-like structure of the TM could not be resolved owing to signal scattering. Several techniques have been employed to reduce optical scattering through different dehydrating agents.^33^^,^^34^ However, addition of chemicals to the eye tissue is not desirable in clinical or research imaging. Given these difficulties in transscleral imaging, we sought to develop a better methodology for imaging the TM.

In this report, we demonstrate the ability of MPM to image deeply into the anterior chamber of an enucleated porcine eye through a gonioscopic lens with image resolution adequate to resolve the collagen fiber bundles of the TM. Porcine eyes were selected because the structure of the surface TM is similar to that found in human eyes.^22^ Collagen fiber bundles (on the order of several microns) can be clearly resolved with our technique. The images presented here show a network of collagen fiber bundles organized into a repeated meshwork of open spaces. The meshwork lacks a clear lamellar organization, similar to that seen by electron microscopy. Unlike the eye of higher primates, the porcine eye has a series of CCs instead of a single large circumferential SC.^22^ We could not detect any of the small CCs deep within the drainage angle of the porcine eye, and we attribute this to both reduced optical power and reduced ability to detect emitted signals at that depth. All the dimensional information of the mesh structures acquired with our multiphoton system (Figs. 2 and 3) agree with our histological measurements (Fig. 4) and those performed by others.^22^

We employed a single plano-convex lens instead of a typical microscope objective to provide the required long working distance to image the porcine TM through the gonioscopic lens. We estimate a working distance of ∼25  mm, numerical aperture of ∼0.21, and magnification of ~7×. This is equivalent to a theoretical image resolution of 2 *μ*m in the lateral direction. This theoretical resolution is calculated based on Abbe’s diffraction limit equation, which assumes no wave-front distortion on the focusing beam.^35^ Owing to the narrow optical bandwidth (∼10  nm) of the excitation pulse and the large detection areas (5 mm in diameter) of the photon-counting detectors, the chromatic aberration that typically hampers the image quality in white light and confocal microscopy is not apparent in our images. The smallest structure that can be resolved in our images is ∼10  μm (collagen fiber bundles of the TM), defining an upper limit of our resolution.

The capability of MPM gonioscopy to image the TM in 3-D with optical resolution adequate to visualize collagen fiber bundles would be of great benefit for obtaining diagnostic information about the health of the TM. MPM gonioscopy could find applications in the clinic and the research laboratory, especially when other technologies do not have the optical resolution to image the detailed mesh structure of the TM. Our current MPM system employs a set of galvo mirrors that scans the TM point by point and requires ∼7.5  s to acquire a single composite image (512×512 pixels). This acquisition speed is certainly not fast enough to overcome small movements associated with *in vivo* imaging in the eye. However, recent technological developments to acquire MPM images with video rate (30  frame/s) have been realized either through a pair of resonant galvo mirrors^36^ or by line-scanning in which temporal focusing^37^ or a high-speed polygonal mirror^38^ were used. Line-scanning schemes potentially have the rapid scanning speed required to overcome tissue movements for *in vivo* eye imaging in the clinic. In particular, the temporal focusing line scanning technique may allow rapid imaging scanning without satisfying the voxel acquisition time required (∼10  μs/voxel) to construct a high signal-to-noise image in the TM.

Recently, 3-D micro-CT has been shown to resolve collagen fiber bundles in an enucleated fixed human eye globe.^11^ In this work, a synchrotron radiation x-ray source was used to achieve 2-*µ*m voxel resolution. The disadvantage of this technique is that synchrotron radiation is generally not available to most laboratories, and the use of fixatives and image contrast agents are literally prohibited to clinical imaging. In comparison, MPM gonioscopy does not need chemical fixation or image contrast agents and requires only a multiphoton laser, a mature technology that is already available to many ophthalmic laboratories and clinics.

Anterior segment OCT (AS-OCT) with spectral-domain data acquisition allows rapid scanning (18,000 to 40,000  A-scan/s) of the anterior chamber and provides a clear picture of the irido-corneal angle.^3^ Although OCT with axial resolution down to 3 *µ*m has been demonstrated in the laboratory,^39^ clinical OCT instruments (including recent developments in phase-sensitive OCT^40^) still do not have the optical resolution and image contrast to reveal the detailed structure of the TM. On the other hand, OCT is very effective in visualizing and measuring the irido-corneal angle of the anterior segment, which is a critical diagnostic parameter in determining the closure of the TM in glaucoma diagnosis.^40^ In this aspect, MPM gonioscopy may be a viable complementary method to OCT to allow close-up inspection of the TM in the clinic.

## 5 Conclusions

The *ex vivo* study presented in this paper is the first demonstration of MPM gonioscopy imaging of the TM region of a porcine eye through a gonioscopic lens. This technique circumvents the highly scattering scleral tissue, thereby achieving higher-resolution images of this important region of the eye. The optical resolution is high enough to resolve detailed collagen fiber structures of the TM. MPM gonioscopy also has the benefit that the emitted signals depend on the intrinsic tissue contrast and not exogenous agents, making it ideal for *in vivo* applications. Future improvement to the optical aberrations of this system can be achieved through compensation tools such as adaptive optics^41^^,^^42^ or application-specific lenses designed using Zemax software (Radiant Zemax, Redmond, Washington) or other ray tracing designs. MPM gonioscopy is a promising imaging modality with potential clinical and research applications aimed at better understanding the regulation of aqueous outflow from the eye, including detection of tissue changes associated with glaucoma as well as monitoring tissue responses to drug and surgical therapies.
